# Genome editing using preassembled CRISPR-Cas9 ribonucleoprotein complexes in *Fusarium graminearum*

**DOI:** 10.1371/journal.pone.0268855

**Published:** 2022-06-03

**Authors:** Nahyun Lee, Jiyeun Park, Jung-Eun Kim, Ji Young Shin, Kyunghun Min, Hokyoung Son

**Affiliations:** 1 Department of Agricultural Biotechnology, Seoul National University, Seoul, Republic of Korea; 2 Research Institute of Agriculture and Life Sciences, Seoul National University, Seoul, Republic of Korea; 3 Department of Microbiology and Immunology, Renaissance School of Medicine, Stony Brook University, Stony Brook, New York, United States of America; Auburn University, UNITED STATES

## Abstract

Genome editing using the clustered regularly interspaced short palindromic repeats (CRISPR)-CRISPR-associated protein 9 (Cas9) system has greatly facilitated the genetic analysis of fungal pathogens. The head blight fungus, *Fusarium graminearum*, causes destructive losses of economically important cereal crops. The recent development of the CRISPR-Cas9 system for use with *F*. *graminearum* has enabled more efficient genome editing. In this study, we described a CRISPR-Cas9-based genome-editing tool for the direct delivery of preassembled Cas9 ribonucleoproteins (RNPs) into the protoplasts of *F*. *graminearum*. The use of RNPs significantly increased both the number of transformants and percentage of transformants in which the target gene was successfully replaced with a selectable marker. We showed that a single double-strand DNA break mediated by the Cas9 ribonucleoprotein was sufficient for gene deletion. In addition, short-homology recombination required only 50 base pair regions flanking the target gene. The high efficiency of Cas9 RNPs enables large-scale functional analysis, the identification of essential genes, and gene deletion that is difficult with conventional methods. We expect that our approach will accelerate genetic studies of *F*. *graminearum*.

## Introduction

The clustered regularly interspaced short palindromic repeats (CRISPR)-CRISPR-associated protein 9 (Cas9) system has been developed into a powerful gene editing method for gene insertion, gene knockout, and gene replacement. The RNA-guided Cas9 endonuclease generates a double-strand break at a DNA target when the target is followed by NGG, the protospacer-adjacent motif sequence. The induced double-strand break can be repaired by either the non-homologous end-joining (NHEJ) or homology-directed recombination (HDR) mechanisms [1]. The NHEJ mechanism tends to repair the DNA incorrectly resulting in short nucleotide insertions and deletions (indels) whereas the HDR mechanism is highly accurate and requires a repair DNA template with a region of homologous sequence [2]. Therefore, the CRISPR-Cas9 system has been widely used to induce or enhance genome mutations in many eukaryotic organisms, including fungi [3].

CRISPR-Cas9-mediated mutagenesis has been widely applied to various filamentous fungal species. In *Trichoderma reesei*, the codon-optimized Cas9 gene was constitutively expressed to induce random mutagenesis [4]. CRISPR expression plasmids were transformed into cells for genome editing in *Neurospora crassa*, *Aspergillus oryzae*, and *Fusarium graminearum* [5–7]. The acceleration of Cas9 expression using the AMA1-based plasmid increases the efficiency of genome editing in *Penicillium chrysogenum* [8]. Moreover, an AMA1-bearing plasmid integrated into the genome could be easily removed because of its great instability [9–11]. However, these methods have practical limitations such as labor-intensive molecular cloning, off-target effects, and prolonged effects of the integrated CRISPR construct.

The use of a Cas9-single guide RNA (sgRNA) complex ribonucleoprotein (RNP) has overcome the limitations of a plasmid-mediated CRISPR-Cas9 system. This expression-free method has several advantages compared to conventional transformation methods. The RNP complex is preassembled *in vitro*; thus, it does not rely on the gene expression mechanism. The RNP is rapidly degraded after genomic DNA cleavage, thereby minimizing off-target cleavage activity and associated toxicity. Recently, RNP transformation has been adapted in various organisms such as plants and animals, including humans [12–14]. With the increasing interest in gene replacement via RNP transformation, there have been several reports of applications to filamentous fungi including *Aspergillus niger* [15], *Fusarium oxysporum* [16], *Penicillium chrysogenum* [17], and *Magnaporthe oryzae* [18]. However, the potential for easy and practical transformation strategy using Cas9 RNP has not been sufficiently demonstrated.

*F*. *graminearum* is an important plant pathogen that causes *Fusarium* head blight in major cereal crops; it also produces mycotoxins in the infected hosts [19]. In this study, we present genomic modification of *F*. *graminearum*, using CRISPR RNP transformation along with a repair construct that contains the desired genome modification. Thus far, homologous gene replacement has been used to generate deletion mutants where transformants can be selected by antibiotic marker genes. Gardiner and Kazan first adapted the CRISPR-Cas9 system in *F*. *graminearum* [7]. However, mutation of the target site is rare in the absence of selection. Based on this previous study, we developed an easy and convenient Cas9-mediated transformation system without labor-intensive molecular cloning. Here, we showed that Cas9 RNP comprised commercially available Cas9 protein; the synthesized sgRNA markedly increased the number of transformants and the gene deletion frequency. Moreover, our CRISPR-Cas9 system enabled an efficient homologous recombination-mediated gene replacement with only 50 base pair (bp) homology arms. This method will facilitate genetic analyses of filamentous fungi including *F*. *graminearum*.

## Materials and methods

### Strains and culture conditions

We used the wild-type (WT) *F*. *graminearum* strain Z-3639; all mutants were derived from the WT strain in this study. The strains were stored as mycelia in 20% glycerol at -80°C. Media used in this study were described in the *Fusarium* laboratory manual [19], and all strains were cultured at 25°C.

### Protein subcellular localization

Protoplasts and conidia were incubated with Cas9 protein fused to green fluorescent protein (GFP) (Applied Biological Materials, Canada) containing the SV40 T antigen nuclear localization sequence (NLS). One hundred fifty microliters of protoplast (5 × 10^5^) and conidium (5 × 10^5^) solutions were incubated with 0.5 μL Cas9 protein (50 pmol/μL; Abcam) for the translocation assay. Samples were incubated at 4°C for an hour. Microscopic observation was achieved using the DM6 B microscope (Leica Microsystems, Wetzlar, Germany), which was equipped with the Leica DMC6200 camera and used the fluorescent filter L5 (Part No. 11504166).

### sgRNA design and in vitro cleavage assay

The sgRNAs were selected where the protospacer adjacent motif sequence (N)_20_NGG was near the 5’ or the 3’ end of the target site. These sgRNAs were checked by a web-based tool CHOPCHOP [20], which could evaluate the off-target potential and GC percentages. Suitable sgRNAs were selected randomly among them. Each synthesized sgRNA was purchased from Macrogen (Seoul, Republic of Korea). The TrueCut™ Cas9 Protein v2 (TrueCut™ Cas9 Protein v2; Thermo-Fisher Scientific, Waltham, MA, USA) was used for the *in vitro* cleavage assay. The 1392 bp PCR fragment was amplified by primers FgPks12/5F and FgPks12 clv-R (Table 1), which contains the target site (S1) that induces cleavage into lengths of 1023 bp and 369 bp. All components were added together in a 1.5-mL microtube up to 10 μL (0.1 ng Cas9 protein, 160 ng sgRNA, 1x Cas9 nuclease reaction buffer, appropriate PCR fragment concentration, and nuclease-free water) and incubated for 30 min at 37°C. Samples were analyzed by gel electrophoresis on a 0.8% agarose gel.

**Table 1:** Primers used in this study.

Name	Sequence	Purpose
FgPks12/5F	CGTTCATGATAACCTCTGTAGTGG	HR donor DNA construction for *PKS12*
pks12-sgRNA1 1kb 5N	GATAACCTACGTTGATCGCTTGG	
pks12-sgRNA2 1kb 3R	AATCGTCGACTACGGGCAAAG	
psk12-sgRNA2 1kb 3N	GCTTGAACGTCATATTGAGGAAAAA	
pks12-sgRNA1 250bp 5F	GACAATCGTGGGGTATCTCTGA	
pks12-sgRNA1 250bp 5N	TGTTGCTCAAGCAGCTAATCCC	
psk12-sgRNA2 250bp 3R	TGATGAAGGGCGTTGATGTAGACT	
pks12-sgRNA2 250bp 3N	GACATGATGCACGGGCGAG	
pks12-sgRNA1 50bp 5F	TAGTTCATTCAACATGACCCCATC	
pks12-sgRNA1 50bp 5N	GGAGGTATTCGTTTTTGGGGAC	
pks12-sgRNA2 50bp 3R	GGCAATCTGCTGCTTCTATCTG	
pks12-sgRNA2 50bp 3N	AATACAAACATCAAAGAAGAATAA	
FgPks12/5R	GCACAGGTACACTTGTTTAGAGAGGAGTAGGTCTTTCAGTGGAGGG	
pks12-sgRNA2 3F	CCTTCAATATCATCTTCTGTCGCCTTGGACAATTAGACACGATGG	
FGSG_04274 5F	TCATTGATGAAGTCGATGCGTAAGA	HR donor DNA construction for *FGSG_04274*
FGSG_04274 5N	TCGTCGGAAACGTCACATACATCT	
FGSG_04274 5R	GCACAGGTACACTTGTTTAGAGCTCCAATGACGACATTACGCTTT	
FGSG_04274 3R	AAGAGCGTGAGGAGGCACTTACA	
FGSG_04274 3N	GTCAAGTTGCACGCATGAATCAGT	
FGSG_04274 3F	CCTTCAATATCATCTTCTGTCGGGCTGGTTATAACACCCTTCAGTCTT	
FGSG_04274 50bp primer R	CTCTTCTTCGTCGCCGATTGGAGGAAGACTGAAGGGTGTTATAACCAGCCCGACAGAAGATGATATTGAA	
FGSG_04274 50bp primer F	ATGAGAGCCAGGAATCATGTCATTTAGAAAGCGTAATGTCGTCATTGGAGCTCTAAACAAGTGTACCTGT	
FGSG_04274 with 5F	TCCCTGTCCTTTGTCCTCATTACC	PCR detection primer
GEN with 5F	GTGTCAGATCAGCCCCACTTGTAG	
neo/G2	GCAATATCACGGGTAGCCAACG	Geneticin tail for fusion
pII99/G3	GGGAAGGGACTGGCTGCTATTG	
FgPks12/5F	CGTTCATGATAACCTCTGTAGTGG	in vitro cleavage assay template
FgPks12 clv-R	GTTCACTCAGTGATGTGGAGCA	
FGSG_04274 5’ seq-F	TTGTCGCGAGCAAGAGAAAAT	FGSG_04274 sequencing primer
FGSG_04274 5’ seq-R	GCCCCTGGGTTCGCAAAG	
FGSG_04274 3’ seq-F	CGCTACTGCTACAAGTGGGGC	
FGSG_04274 3’ seq-R	GAAGTTAAACCTCGGGAATGAAG	

### Fungal transformation

To generate deletion mutants, the 5’ and 3’ flanking regions of *FgPKS12* and *FGSG_04274*, as well as the geneticin resistance gene cassette (*GEN*), were amplified from the genomic DNA WT stain Z-3639 and plasmid pII99, respectively. The double-joint PCR method [21] was performed to construct the fusion PCR products; the resulting amplicons were used for transformation into the WT strain. The constructed short-homology-mediated transformation donor DNAs were amplified by the primers FGSG_04274 50bp primer R and FGSG_04274 50bp primer F (Table 1). These primers were designed to contain 20 bp of the *GEN* cassette with 50 bp of sequence upstream and downstream of the open reading frame (ORF) of the target gene. The amplified PCR constructs were directly used as donor DNAs for gene replacement with the RNP complexes required in the protocol. For fungal transformation, 150 μL fungal protoplasts (5 × 10^5^) were mixed with 10 μL Cas9 RNP complexes and donor templates for gene integration. After incubation in polyethylene glycol for 15 min, 1 mL STC buffer (1.2 M sorbitol, 10 mM CaCl_2_, 10 mM pH 7.5 Tris-HCl) was added to the mixture. The other fungal transformation procedures were performed as previously described [22].

### DNA extraction and Southern blotting

Genomic DNA was extracted from freeze-dried mycelial powder as described [19]. Standard protocols were used for restriction endonuclease digestion, agarose gel electrophoresis and Southern blotting [23].

## Results and discussion

### Nuclear localization of the commercial Cas9 protein in F. graminearum

**Fig 1:**
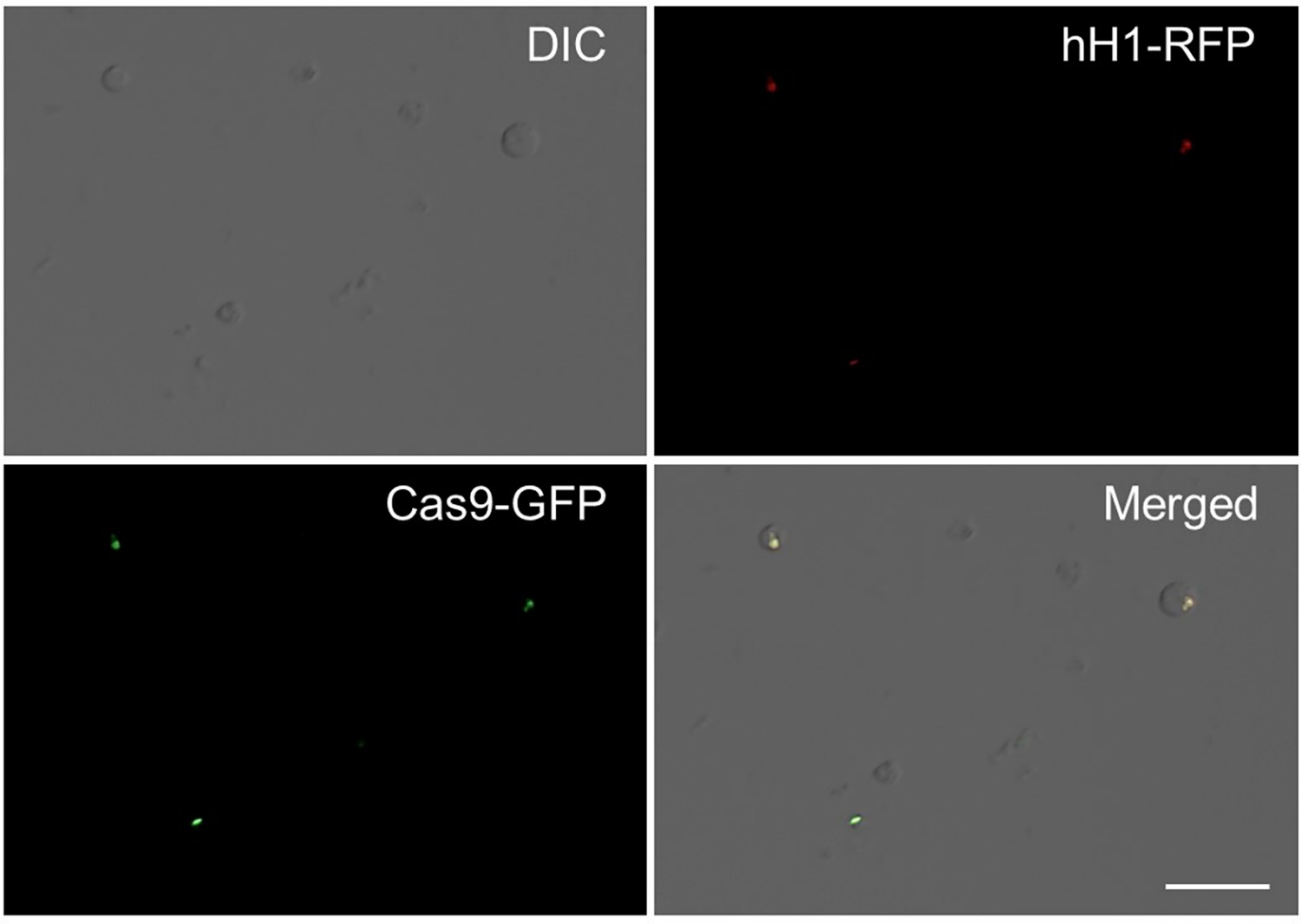
Subcellular localization of Cas9-eGFP protein in *F*. *graminearum*. GFP fluorescence was colocalized with histone H1-RFP in protoplasts. The results indicated that SV40 NLS is functional for the nuclear localization of Cas9 protein in this fungus. Scale bars, 20 μm.

The subcellular localization of the commercial Cas9 protein was investigated to determine the optimal Cas9 for *F*. *graminearum*. Commercial Cas9 proteins generally contain an NLS [24] to enable protein translocation into the nucleus where genome editing occurs. In *F*. *oxysporum*, a previous study showed that the SV40 NLS is not functional for the nuclear localization of Cas9; therefore, endogenous histone H2B NLS was utilized for the successful translocation of Cas9 proteins into nuclei [16]. In this study, we assessed whether SV40 NLS is sufficient for the nuclear localization of Cas9 protein in *F*. *graminearum* (Fig 1). For colocalization analysis, Cas9-eGFP containing SV40 NLS was incubated with protoplasts of the *F*. *graminearum* hH1-RFP strain, in which histone H1 was fused to red fluorescent protein (RFP) [25]. We found that GFP fluorescence was exclusively present in the nuclei of the *F*. *graminearum* protoplasts, suggesting that protoplasts can be used for RNP-mediated fungal transformation.

### *In vitro* nuclease activity of the preassembled Cas9 RNP

**Fig 2:**
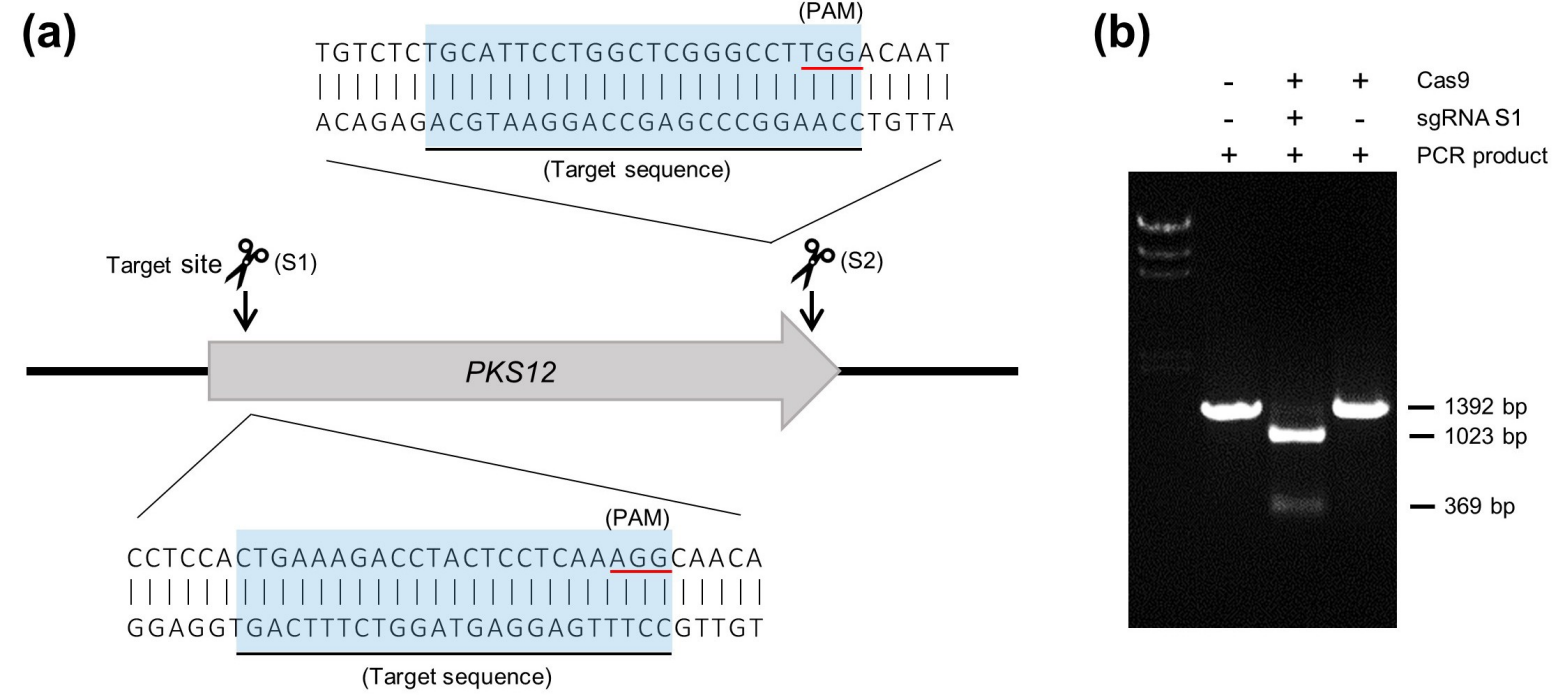
Schematic representation of the *in vitro* cleavage assay of preassembled Cas9 RNP. (a) Schematic representation of sgRNA design for *in vitro* cleavage assay and preassembled Cas9 RNP-mediated transformation. The two sgRNAs were designed to cleave two sites, S1 (49–71 bp) and S2 (6433–6455 bp), respectively. The protospacer adjacent motif sequence is required for Cas9 to recognize the target site. (b) *In vitro* cleavage assay. The results showed the activity of preassembled Cas9 RNP.

The purpose of our experiment was to validate the efficiency of the Cas9 RNP-mediated gene deletion system in *F*. *graminearum*. First, we examined the endonuclease activity of the preassembled Cas9 RNP. The *PKS12* gene was targeted because the *pks12* mutations result in a visible albino phenotype, thus simplifying their identification (Fig 2A) [26]. We designed two sgRNAs, which target both ends of the Pks12-coding sequence near the start and stop codons. To predict the possibility of off-targeting, the CHOPCHOP web-based tool for identifying CRISPR/Cas9 off-target sites [20] was used to evaluate mismatches with each genomic site (Table 2). The nuclease activity of the preassembled Cas9 RNP was tested using an *in vitro* cleavage assay. The 1392 bp linear DNA segment included a target cleavage site, and the DNA fragments were designed to be 1023 bp and 369 bp (Fig 2B). The linear DNA was incubated with the preassembled Cas9 RNP comprising Cas9 and the sgRNA S1. The preassembled Cas9 RNP efficiently cleaved the linear DNA into two expected sizes of DNA fragments.

**Table 2:** The CHOPCHOP [20] results of selected sgRNAs. The number of potential off-targets and GC% of the target sequence are shown.

Target gene	Target sequence (sgRNA labeling)	Strand	GC content (%)	Off-targets		
				MM1	MM2	MM3
*PKS12*	CTGAAAGACCTACTCCTCAAAGG (S1)	-	45	0	0	0
	TGCATTCCTGGCTCGGGCCTTGG (S2)	-	65	0	0	0
*FGSG_04274*	GTTAAGCAGTTCTAGCGGGCTGG (S3)	-	55	0	0	0
	TAATGTCGTCATTGGAGCGCCGG (S4)	-	50	0	0	0

### Construction of *PKS12* deletion mutants via RNP-mediated transformation

**Fig 3:**
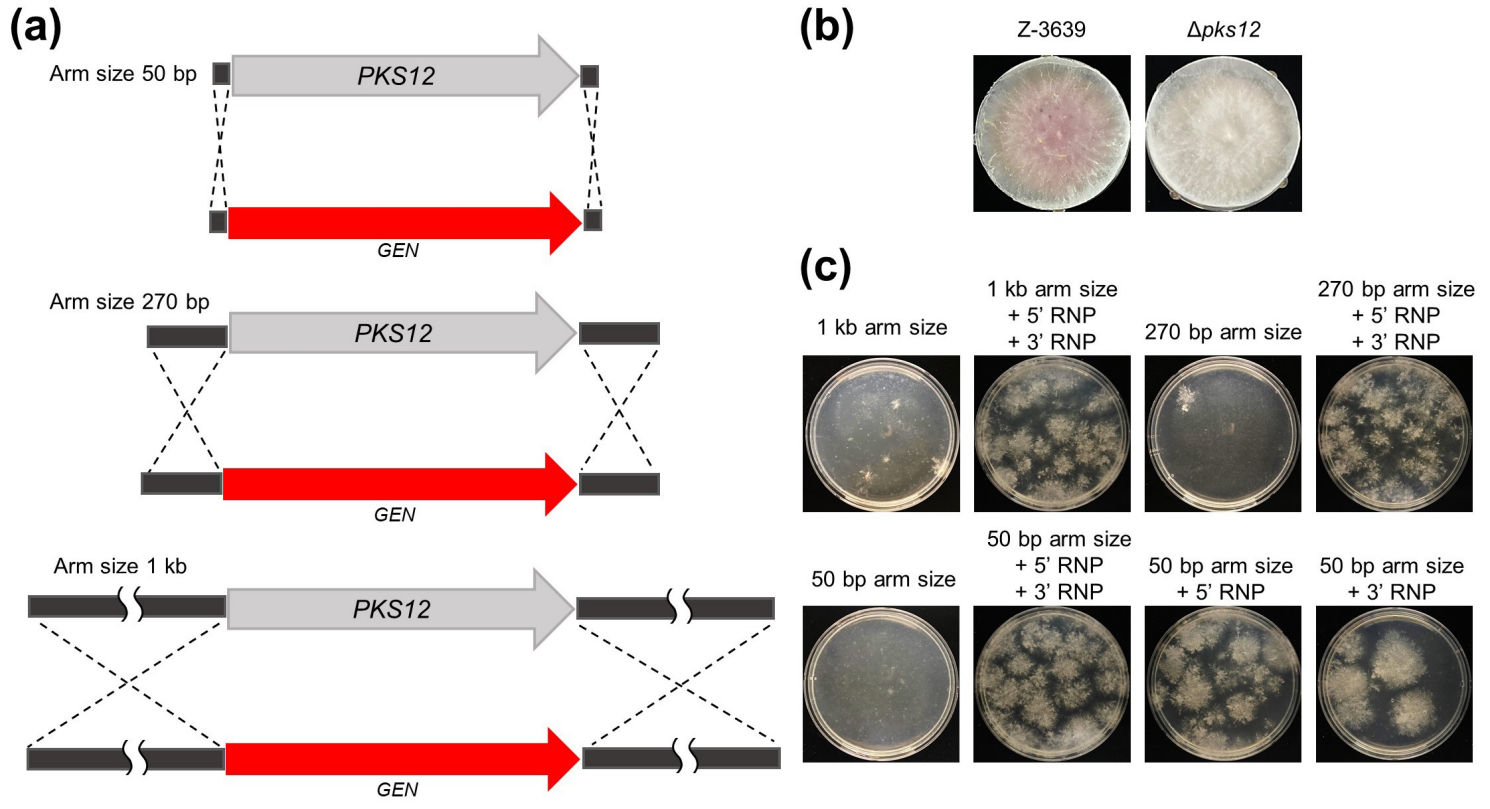
Construction of deletion mutants by Cas9 RNP-mediated transformation with different homology arm sizes. (a) Schematic representation of *PKS12* deletion. Three constructs with different homology arm sizes (1 kb, 270 bp, and 50 bp) were generated for *PKS12* deletion. (b) Colony morphologies of *F*. *graminearum* strains generated during Cas9 RNA-mediated transformation. (c) Each randomly chosen plate of disrupted phenotypes was counted as transformants in regeneration media. Expected phenotypes were later calculated as frequencies.

In *F*. *graminearum*, transformation for genetic manipulation is generally achieved by the integration of a selectable marker gene via homologous recombination [27, 28]. Here, the deletion construct containing the *GEN* for selection was synthesized by the double-joint PCR strategy [21] with three different homology arm sizes (1 kb, 270 bp, and 50 bp) (Fig 3A). After transformation with or without RNPs, *PKS12* deletion was identified by the albino phenotype because Δ*pks12* mutants could not produce the red pigment aurofusarin (Fig 3B).

When deletion constructs with shorter homology arm size were used for fungal transformation without RNPs, both the transformant numbers and deletion efficiencies were markedly decreased in *F*. *graminearum* (Table 3 and Fig 3C). In the absence of the RNP, only 5.5% of the colonies were identified as Δ*pks12* mutants when the deletion construct had 270 bp homology arms. No deletion mutant was produced when the deletion construct with 50 bp homology arms was transformed without the RNP. In the presence of the RNP, however, the transformant number increased by at least 10-fold, and the deletion efficiency was significantly higher. Notably, the transformant number and deletion efficiency were not dependent on the arm sizes when the RNPs were used. This suggests that the 50 bp homology arm size is sufficient for homology-directed recombination in *F*. *graminearum*. We examined whether a single RNP with 50 bp homology arms could induce gene deletion (Table 3). The transformation frequencies and deletion efficiencies were different when sgRNA S1 or sgRNA S2 was used for the RNP. Although sgRNA S2 showed a lower yield than did S1, it was sufficient for target gene deletion (Table 3 and Fig 3C).

**Table 3:** Deletion efficiencies were lower when the homologous arm size of the construct was shorter. Because of variation in the transformation results, this table shows a representative experiment.

Target gene	Arm size	sgRNA	Total no. of transformants	Expected phenotype frequency	Deletion efficiency (%)
*PKS12*	1 kb	S1 + S2	276	5.5 × 10^−4^	91.7
*PKS12*	1 kb	-	28	5.6 × 10^−5^	22.9
*PKS12*	270 bp	S1 + S2	296	5.9 × 10^−4^	95.8
*PKS12*	270 bp	-	18	3.5 × 10^−5^	5.5
*PKS12*	50 bp	S1 + S2	316	6.4 × 10^−4^	95.8
*PKS12*	50 bp	-	4	8.0 × 10^−6^	0
*PKS12*	50 bp	S1	222	4.4 × 10^4^	95.8
*PKS12*	50 bp	S2	44	8.8 × 10^5^	63.6

### Confirmation of short-homology-mediated integration as an efficient gene deletion strategy

**Fig 4:**
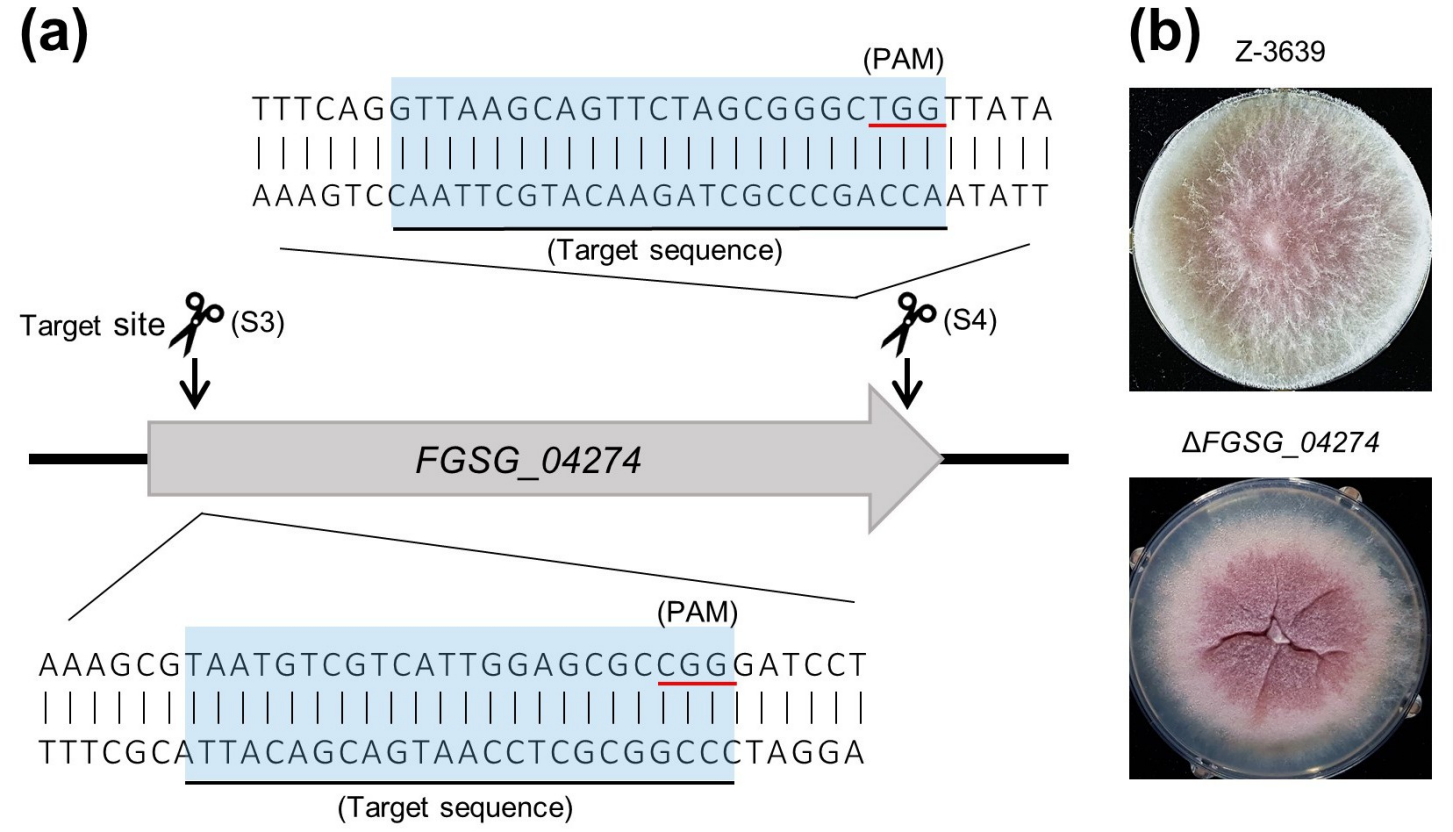
An identical deletion strategy was applied to *FGSG_04274* to demonstrate the replication of deletion efficiencies via RNP complexes. (a) Schematic representation of sgRNA design for preassembled Cas9 RNP-mediated transformation. The two sgRNAs were designed to cleave two sites, S3 (18–40 bp) and S4 (1093–1115 bp). (b) Deletion mutants were identified by the dark red color and abnormal shapes of the colonies.

To determine whether Cas9 RNP would allow gene deletion for loci other than *PKS12*, we used the system to delete the uncharacterized gene *FGSG_04274*. The deletion mutants have an abnormal colony morphology (i.e. defective vegetative growth), thus simplifying their identification (Fig 4B). Two sgRNAs were designed to target near the start codon (sgRNA S3) and the stop codon (sgRNA S4) (Table 2 and Fig 4A). As expected, transformation without RNP resulted in poor deletion efficiencies, compared to transformation with the RNPs (Table 4).

**Table 4:** High efficiency of gene deletion via RNP transformation was tested by *FGSG_04274* for verification. Deletion mutants were identified by the dark red color and abnormal shapes of the colonies. *50 bp indicates that *GEN* is amplified by 70 bp primers, which contain 50 bp arms homologous to sequences upstream or downstream from *FGSG_04274*.

Target gene	Arm size	sgRNA	Total no. of transformants	Deletion efficiency (%)
*FGSG_04274*	1 kb	S3 + S4	652	97.2
*FGSG_04274*	1 kb	S3	56	72.2
*FGSG_04274*	1 kb	S4	579	97.2
*FGSG_04274*	1 kb	-	12	58.3
*FGSG_04274*	* 50 bp	S3 + S4	435	93.8
*FGSG_04274*	* 50 bp	S3	74	87.5
*FGSG_04274*	* 50 bp	S4	262	91.7
*FGSG_04274*	* 50 bp	-	7	0

We designed a gene deletion construct that had 50 bp arms homologous to sequences upstream or downstream from the target gene. This construct can be synthesized with chimeric primers using a simple PCR method, thus minimizing time and financial cost for researchers. The *GEN* selection marker was amplified with 70 bp primers, which included 50 bp homology arms to the target gene *FGSG_04274* (Table 1). In the absence of the RNP, the transformation produced few colonies but did not show abnormal colony morphology (Table 4). However, short-homology-mediated integration of the donor gene coupled with the RNP markedly increased transformant frequency and deletion efficiency. We also observed that PCR genotyping could successfully detect randomly chosen transformants, which were presumably deletion mutants (S1 Fig). Moreover, deleted regions were confirmed by Southern blot and sequence analysis (S2 Fig).

In this study, we established an efficient gene deletion approach using the Cas9 RNP and a deletion construct with various homology arm sizes. An important benefit of this approach is the ability to use commercially available Cas9 protein and custom-synthesized RNAs. Therefore, no additional laboratory equipment or techniques are required for CRISPR expression; only the deletion construct must be synthesized using PCR. Through our RNP transformation approach, we expect that challenging genetic modifications can be more effectively applied to filamentous fungi, including *F*. *graminearum*. The ability to transform protein and RNA into *F*. *graminearum* implies the potential for transforming other proteins such as enzymes, biosensors, or inhibitory proteins.

## Supporting information

S1 Raw images (PDF)

S1 Fig PCR detection strategy for FGSG_04274 and Δ*FGSG_04274*::*GEN*. Five abnormal phenotype transformants and a wild-type-like transformant were randomly chosen. The left electrophoresis band indicates the amplified ORF by internal primers and the right band is for detecting deletion mutants that are amplified by primer included in *GEN*. Each transformants was detected linearly.
(TIF)

S2 Fig Confirmation of gene replacement. (a) Three Δ*FGSG_04274* transformants and a wild-type-like transformant were chosen for Southern blotting. (b) Sequence analysis was performed by Bioneer (Seoul, Republic of Korea) and the marker gene fragments were integrated as designed.
(TIF)
