# Supplementary figures and images for: Genome editing using preassembled CRISPR-Cas9 ribonucleoprotein complexes in Fusarium graminearum
Source: PLoS One. 2022 Jun 3;17(6):e0268855. doi: 10.1371/journal.pone.0268855 (PMC9165886; doi:10.1371/journal.pone.0268855)

## S1 Raw images:

### 1. Raw image of Fig 2b.

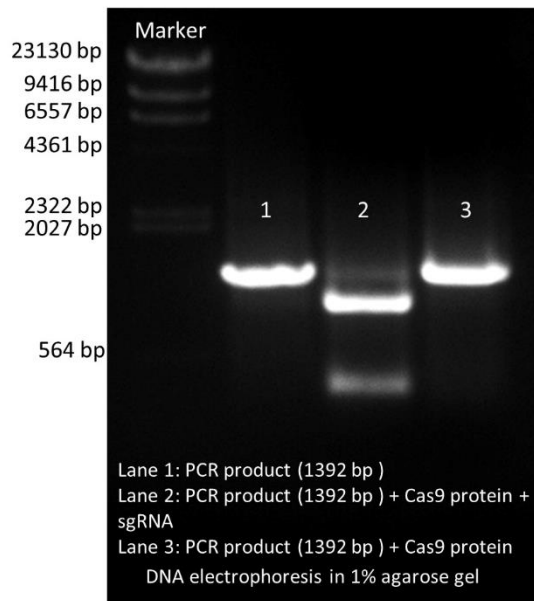

### 2. Raw image of S2 Fig.

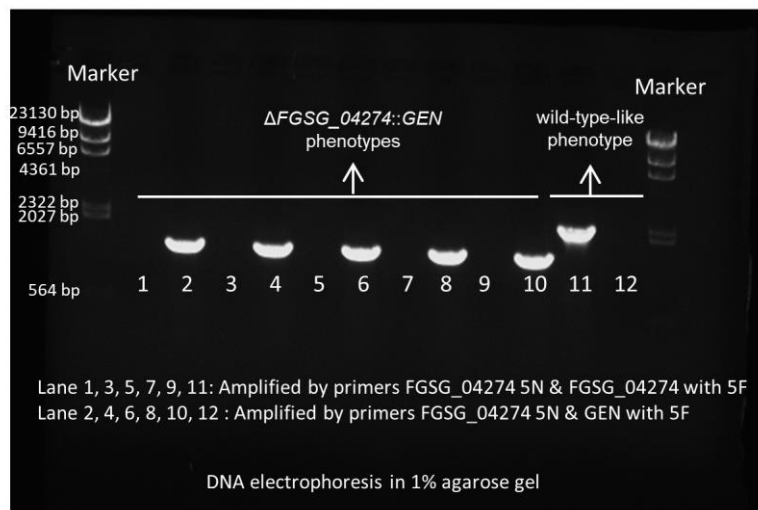

### 3. Raw image of S3 Fig a.

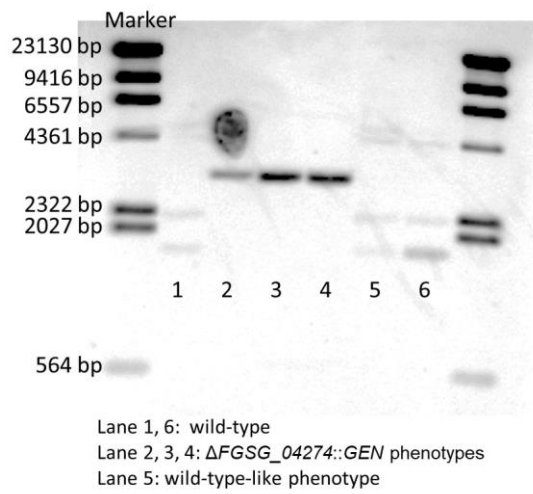

Supplement: S1 Raw images — (PDF) [file pone.0268855.s001.pdf]

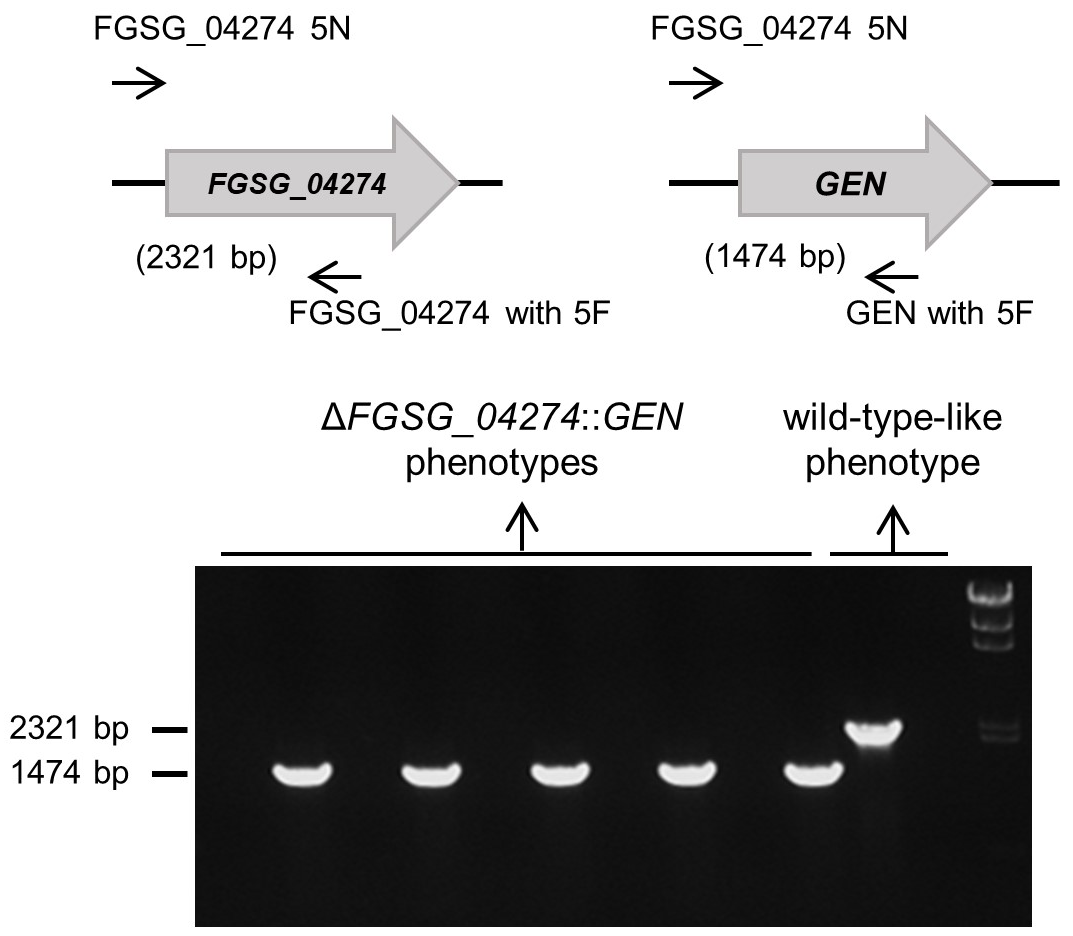

Supplement: S1 Fig — Five abnormal phenotype transformants and a wild-type-like transformant were randomly chosen. The left electrophoresis band indicates the amplified ORF by internal primers and the right band is for detecting deletion mutants that are amplified by primer included in GEN. Each transformants was detected linearly. (TIF) [file pone.0268855.s002.tif]

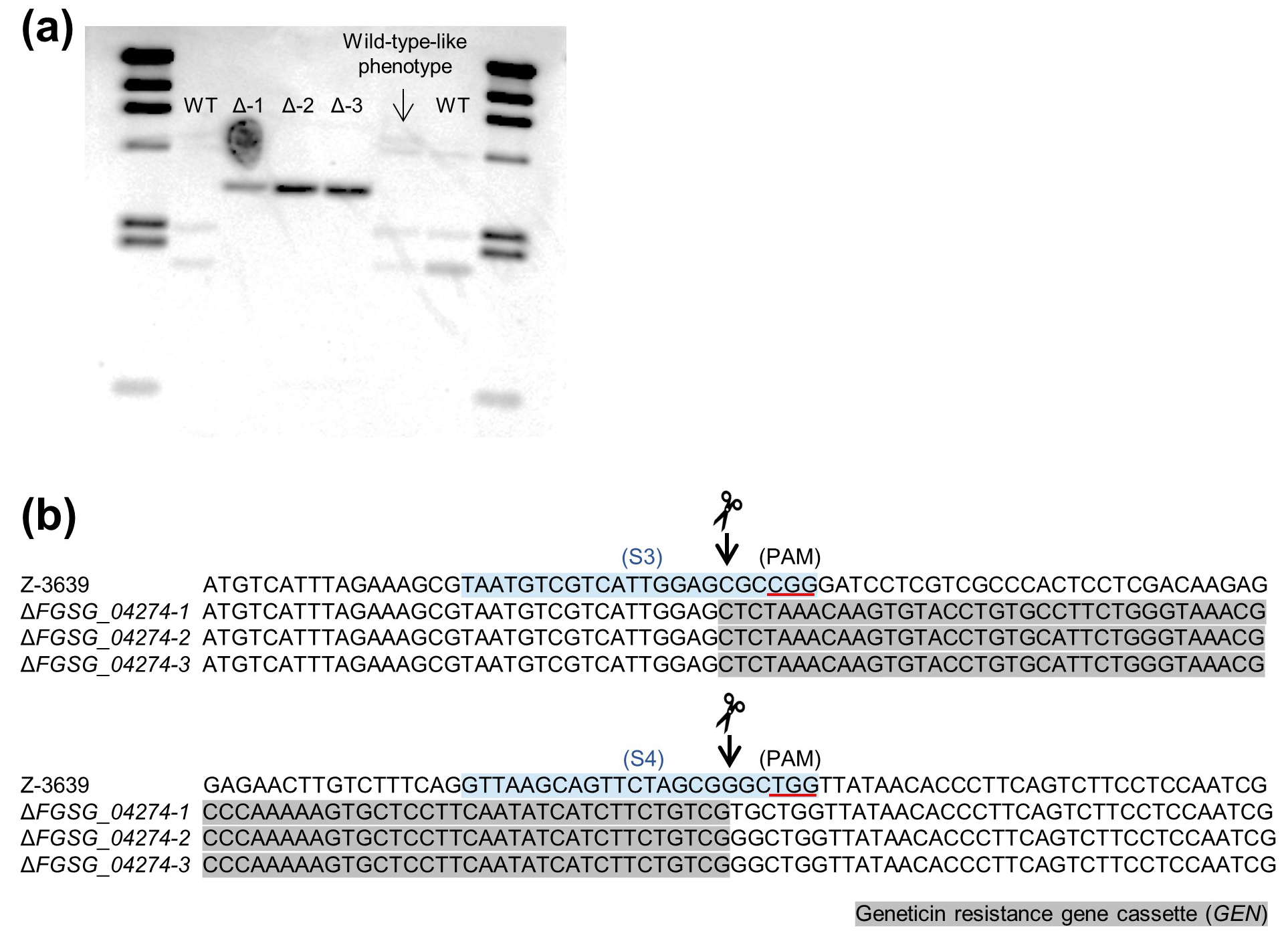

Supplement: S2 Fig — (a) Three ΔFGSG_04274 transformants and a wild-type-like transformant were chosen for Southern blotting. (b) Sequence analysis was performed by Bioneer (Seoul, Republic of Korea) and the marker gene fragments were integrated as designed. (TIF) [file pone.0268855.s003.tif]
